# The discovery of a novel inhibitor of apoptotic protease activating factor-1 (Apaf-1) for ischemic heart: synthesis, activity and target identification

**DOI:** 10.1038/srep29820

**Published:** 2016-07-22

**Authors:** Ying Wang, Yang Cao, Qing Zhu, Xianfeng Gu, Yi Zhun Zhu

**Affiliations:** 1 Department of Pharmacology, School of Pharmacy, Fudan University, Shanghai 201203, China; 2 Department of Medicinal Chemistry, School of Pharmacy, Fudan University, Shanghai 201203, China; 3 School of Pharmacy, Nantong University, Nantong, 226001, China; 4 School of Pharmacy, Macao University of Science and Technology, Macao

## Abstract

Apaf-1 is a central component in the apoptosis regulatory network for the treatment of apoptosis related diseases. Excessive Apaf-1 activity induced by myocardial ischemia causes cell injury. No drug targeted to Apaf-1 for treating myocardial ischemia has been reported to the best of our knowledge. In the present work, we synthesized a novel compound, **ZYZ-488**, which exhibited significant cardioprotective property in significantly increasing the viability of hypoxia-induced H9c2 cardiomyocytes and reducing CK and LDH leakage. Further study suggested the protective activity of **ZYZ-488** dependent on its anti-apoptosis effect. This anti-apoptotic effect is most probably related to its disturbing the interaction between Apaf-1 and procaspase-9 as the target fishing and molecular docking indicated. The suppression on the activation of procaspase-9 and procaspase-3 with **ZYZ-488** strongly suggested that compound **ZYZ-488** could be a novel inhibitor of Apaf-1. In conclusion, **ZYZ-488** as a novel small molecule competitive inhibitor of Apaf-1, with the great potential for treating cardiac ischemia.

Leonurine (**LEO**, also named as **SCM-198**) is a natural alkaloid chemically synthesized by our laboratory from *Herba leonuri* which has long been used in chinese traditional medicine. The studies on **LEO** show that it has cardio^1^,^2^ and neuronal^3^ protective effects both *in vitro* and *in vivo*. The evidence in animal studies of **LEO** exhibits cardioprotective effects on myocardial ischemia disease in rats after oral administration. Therefore, **LEO** has been considered as a potential drug candidate in our laboratory for further studies. However, pharmacokinetic studies showed that the major of **LEO** was quickly metabolized into leonurine-10-O-ß-D-glucuronide (**ZYZ-488**, Fig. 1) after oral adminstration. The level of **ZYZ-488** was far higher (approximately 20-fold) than its parent drug in the pooled plasma samples after oral administration at 30 mg/kg^4^. To fully understand the mechanism of action of drugs, it is important to recognize the role of major metabolites^5^. Thus, the studies on the synthesis and the biological activity of **ZYZ-488** has been further performed.

The previous studies of our group show that **LEO** exhibited protective effects in myocardial hypoxia, especially in anti-apoptotic assays^1^,^3^,^6^. As we known, apoptosis is a physiological counterpart of cell replication and is the contributing cause to cardiomyocyte death during ischemia, myocardial infraction (MI) and heart failure^7^. Several reports have shown that ischemia/reperfusion-induced infarct extension can be significantly attenuated by anti-apoptotic therapy. However, apoptotic signaling networks are very intricate, regulated by a fine-tuned balance between pro-apoptotic and anti-apoptotic proteins^8^. Different cellular pathways can lead to apoptosis. For example, in the presence of ATP/*d*ATP, the apoptosome is assembled when seven Apaf-1; cyto-chrome c heterodimers oligomerise to form a ‘wheel’ structure that has the ability to recruit procaspase-9. The cytochrome c binds the adapter molecule Apaf-1 and, it promotes the assembly of a multiproteic complex called apotosome which, in turn, binds and activates procaspase-9, the main constituent of the apoptosome is Apaf-1, Apaf-1 is a multidomain protein with an N-terminal caspase recruitment domain (CARD), a central nucleotide-binding and oligomerization domain (NOD), and a C-terminal WD40 repeats domain^9^. Apoptosis protease-activating factor-1 (Apaf-1) has been described as a key regulator of the mitochondrial apoptosis pathway^6^ and considered an emerging pharmacological target. However, only few compounds targeting it have been tested and none of these have yet been approved by FDA for starting clinical trials. Therefore, it is imperative to develop more and new Apaf-1 inhibitors. Coincidentally, the studies on target fishing and molecular docking discovered **ZYZ-488** most probably binding to Apaf-1 in procaspase-9 binding site. As expected, western blot confirmed **ZYZ-488** inhibited the activation of binding protein procaspase-9, while not tampering the expression of Apaf-1, and studies on target specificity by siRNA based approaches further proved **ZYZ-488** is a novel inhibitor of Apaf-1. These studies provide us a fresh insight for agent design and modification in treating ischemia.

## Results

### Chemistry

**Figure 2:**
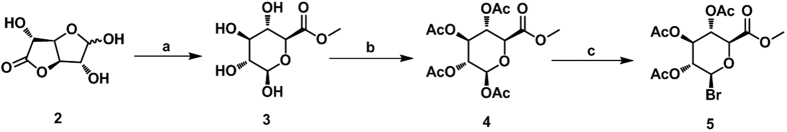
The synthetic route of key intermediate 5. (**a**) CH_3_OH, NaOH, r.t., 3 h; (**b**) Ac_2_O, HClO_4_, r.t., 12 h; (**c**) CH_3_OH, HBr, N_2_, 0 °C, 2 h.

**Figure 3:**
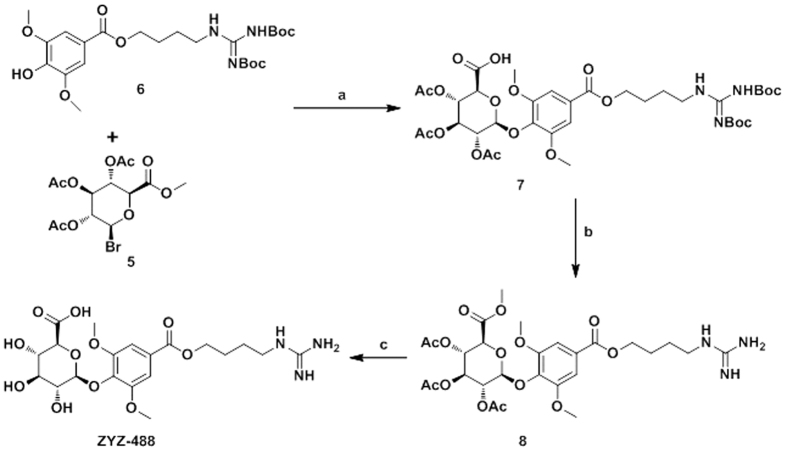
The synthetic route of compound ZYZ-488 . (**a**) CHCl_3_, TBAB, K_2_CO_3_, 12 h; (**b**) CH_2_Cl_2_, TFA, r.t., 12 h; (**c**) CH_2_Cl_2_/CH_3_OH (1:9), guadine, 0 °C, 2 h.

The synthesis of compound **ZYZ-488** and key intermediate **5** is outlined in Figs 2 and 3. The preparation of key intermediate was carried out starting from glucurolactone (**2**). After treatment of NaOH and methanol, the resulting methyl ester **3** was acetylated by Ac_2_O and HClO_4_ to afford the intermediate **4**. Subsequently, **4** was treated with HBr in acetic acid to give desired intermediate **5**.

To prepare target compound **ZYZ-488**, intermediate **5** condensed with another key intermediate **6** which was prepared as previously described^10^, to afford **7**. Finally, the Boc groups in compound **7** were removed by trifluoroacetic acid (TFA) followed by hydrolyzation by guadine to give target compound **ZYZ-488**.

### Protective effects of compound ZYZ-488 on hypoxia-induced H9c2 rat ventricular cells

**Figure 4:**
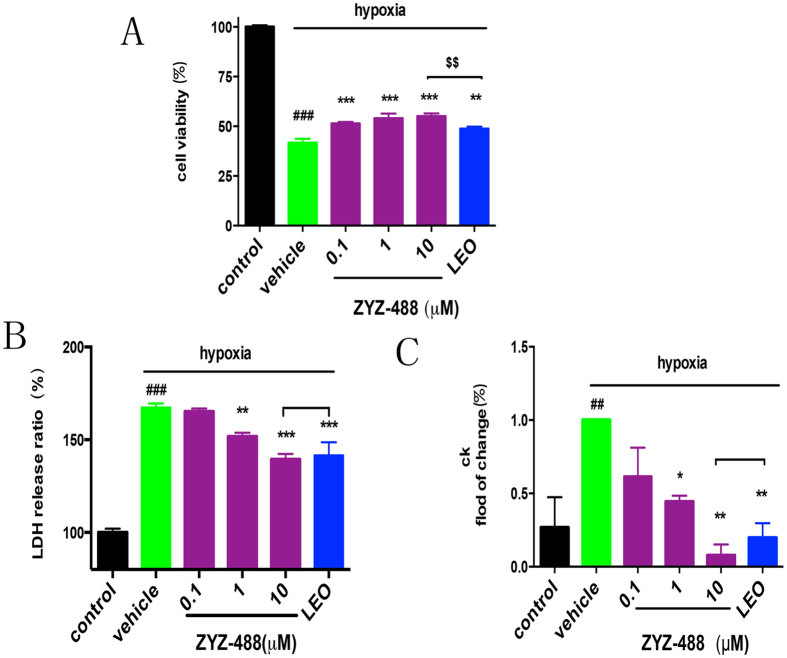
Analysis of the cardioprotective activities of ZYZ-488 and LEO (10 μM) in hypoxia-induced H9c2 rat ventricular cells. (**A**) Effects of different dosed **ZYZ-488** and **LEO** on cell viability in hypoxia-induced H9c2 rat ventricular cells. (**B**) Effects of **ZYZ-488**, **LEO** on LDH leakage in hypoxia-induced H9c2 rat ventricular cells. (**C**) Effects of **ZYZ-488**, **LEO** on CK activity in hypoxia-induced H9c2 rat ventricular cells. H9c2 cells were treated with **ZYZ-488**, **LEO** in hypoxia for 12 h. ^*#*^*P* < 0.05; ^*##*^*P* < 0.01; ^*###*^*P* < 0.001 versus control group. ^*^P < 0.05; ^**^P *<* 0.01; ^***^P < 0.001 versus vehicle group. ^$$^*P* < 0.01 versus **LEO** 10 μM treatment group. Error bars represent the data obtained from the experiments repeated three times or more.

The synthetic **ZYZ-488** and **LEO** were evaluated on their protective effects against hypoxia in H9c2 rat ventricular cells. The effects of compound **ZYZ-488**, and **LEO** were investigated by cell counting kit-8 (CCK8) assay which demonstrates cell viability. It showed that the hypoxic condition had an effect on H9c2 rat ventricular viability compared with the normoxic control group (*P* < 0.001). Cells were treated with 0.1, 1, and 10 μM of **ZYZ-488** and 10 μM of **LEO** during hypoxia for 12 hours. Treatment of **ZYZ-488** led to increase in the number of surviving cells at a concentration-dependent manner [0.1 μM (51.46 ± 7.42)%, 1 μM (54.15 ± 2.26)%, 10 μM (55.19 ± 1.28)%] compared to vehicle group (41.76 ± 1.90). Most interestingly, 10 μM of compound **ZYZ-488** showed stronger activity on promoting the cell vitality compared with the group treated by **LEO** in same concentration (*P* < 0.01) (Fig. 4A). Lactic dehydrogenase (LDH) leakage (percentage of normoxic control group) into the culture medium after the induction of hypoxia was significantly higher than that in the normoxic control group [(167.37 ± 2.20)% vs (100%)]. Compared with the vehicle group, 1 μM and 10 μM of compound **ZYZ-488** and 10 μM of **LEO** remarkably inhibited LDH leakage into medium which is the marker of cell membrane integrity^11^ (*P* < 0.05) (Fig. 4B). Elevated Creatine Kinase (CK) is a canonical index of myocardial infarction in clinical diagnosis. Hypoxia caused cell injury, thus resulting in increased CK leakage into the culture medium^12^ than the normoxic group [(100)% vs (26.8 ± 20.64) %]. In our experiment, CK leakage was significantly reduced when cardiomyocytes were treated with **ZYZ-488** at 1 μM [(44.49 ± 3.92)%] and 10 μM [(7.848 ± 7.39)%] and **LEO** at 10 μM [(19.75 ± 9.93%)](Fig. 4C), suggesting that **ZYZ-488** was more potent than **LEO** in increasing cell viability against hypoxia (*P* < 0.01). Thus, the results above confirmed that **ZYZ-488** exhibited significant, even more potent cardioprotective activity than **LEO** in hypoxia-induced H9c2 cells injury.

### Compound ZYZ-488 decreased apoptosis in hypoxic H9c2 cells

**Figure 5:**
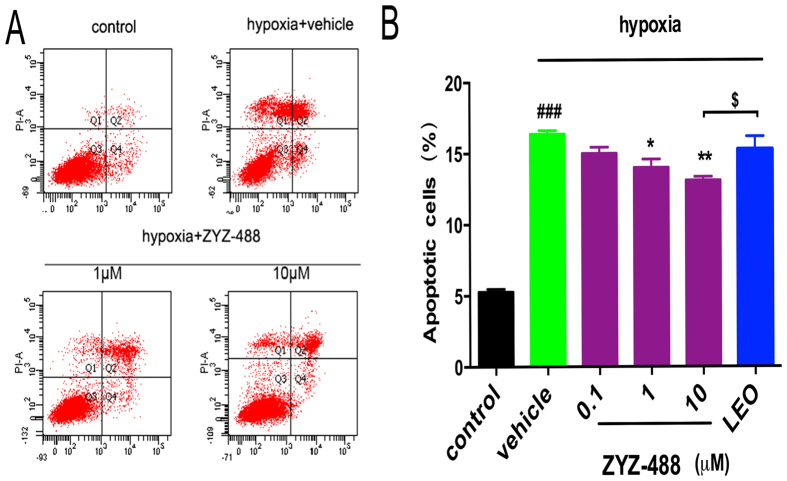
(**A**) Apoptosis fraction of hypoxia-induced H9c2 rat ventricular cells with or without **ZYZ-488** and **LEO** was measured by AnnexinV-FITC/PI assay. (**B**) Percentage of apoptotic cardiomyocytes under the treatment of **ZYZ-488** and **LEO** was then analyzed based on AnnexinV-FITC/PI stain. ^*###*^*P* < 0.001 versus control group. ^*^P < 0.05; ^**^P *<* 0.01; ^***^P < 0.001 versus vehicle group. ^$^*P* < 0.01 versus **LEO** 10 μM treatment group.

**Figure 6:**
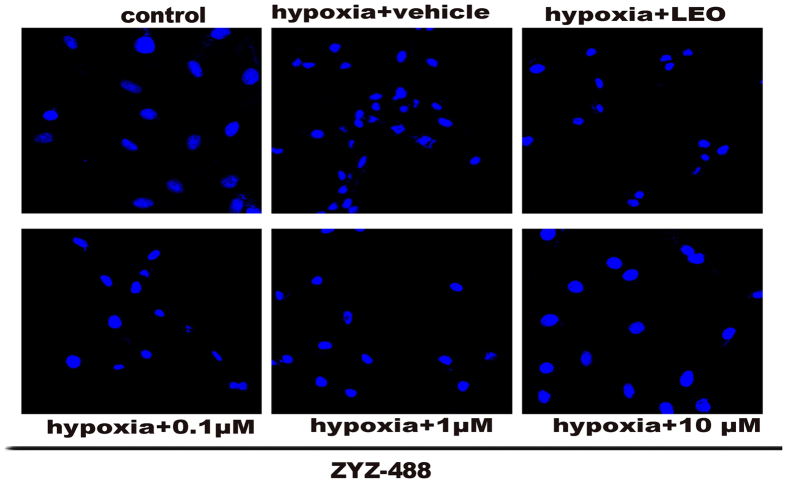
Hoechst staining H9c2 cells exposed to normoxia or hypoxia for 12 h in the absence or presence of ZYZ-488 and LEO, H9c2 cells apoptosis were observed by confocal microscopy.

Apoptosis is responsible for a significant amount of the cardiomyocyte death that contributes to the development and progression of heart failure^13^. It has been shown that hypoxia represents the most physiologically relevant stresses lead to rapid apoptosis^14^,^15^,^16^. The apoptotic cell counts were measured using AnnexinV-FITC/PI by flow cytometry. The results displayed that **ZYZ-488** and **LEO** decreased the number of apoptotic cells (Fig. 5A). The percentage of cells showing an apoptotic pattern (including early apoptotic and late apoptotic cells) was significantly decreased with **ZYZ-488** at 1 μM [(14.00 ± 0.59)%], 10 μM [(13.1 ± 0.26)%] and **LEO** at 10 μM [(15.28 ± 0.92)%] compared with vehicle group [(16.38 ± 0.13)%] (Fig. 5B). Imaging of treated cells was done using a confocal microscope. Marked cellular morphological characteristics of apoptosis, such as condensation of chromatin and nuclear fragmentation, were clearly seen using Hoechst 33258 staining (Fig. 6). As demonstrated above, **ZYZ-488** is potent in protecting myocardial cells from hypoxia injury. According to the anti-apoptotic results, we speculate **ZYZ-488** elicits cardioprotective effects through its anti-apoptotic activity.

### Prediction of molecular target of compound ZYZ-488

**Figure 7:**
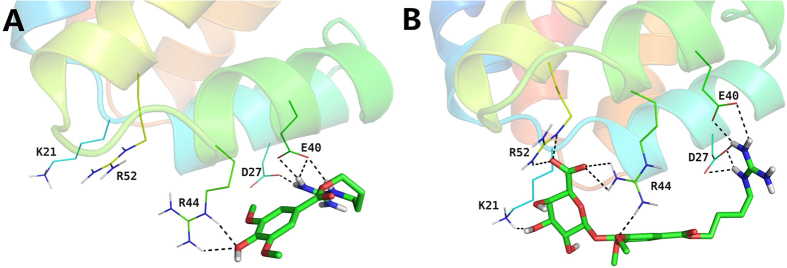
Overlapped poses of **LEO** (**A**) and compound **ZYZ-488** (**B**) at the procaspase-9 binding site of Apaf-1. The ligands are shown in sticks. The coloring for oxygen atoms of each compound is red. The hydrogen bonds are denoted with dashed lines. Structure figures were prepared using LigPrep (Schrödinger Release 2015-4: LigPrep, version 3.6, Schrödinger, LLC, New York, NY, 2015).

Cell apoptosis involves complex signaling network. To identify potential targeting candidates of compound **ZYZ-488**, *in-silico* targets screening was conducted. PharmMapper server, a reverse pharmacophore mapping approach was performed using an in-house pharmacophore database (PharmTargetDB)^17^. Apoptotic protease-activating factor 1 (Apaf-1), a key regulator of the apoptosis machinery^18^, is in the top 0.3% of prediction results. Combining with our results of anti-apoptotic activity of **ZYZ-488**, we speculated that compound **ZYZ-488** might interact with Apaf-1 to suppress the apoptosis, then elicit the protective effect on H9c2 cells. It has been established that binding of caspase recruitment domain (CARD) of Apaf-1 to procaspase-9 leads to apoptotic cell death^19^. X-ray crystal structure of the complex of Apaf-1 CARD binding with the procaspase-9 prodomain (PDB code: 3YGS) has been determined at2.5 Å resolution by Shi *et al*.^20^. Three arginine residues (Arg 13, Arg 52, and Arg 56) from procaspase-9 prodomain were identified to play a vital role in the stability of the Apaf-1 CARD/procaspase-9 prodomain complex. Hydrogen bonds network could be observed between these three arginine residues and Asp 27/Glu 40 from Apaf-1 CARD^20^. In view of the common basic guanidyl groups, leonurine and **ZYZ-488** may be capable of binding to the acidic residues of Apaf-1 CARD as mimics of Arg 13/Arg 52/Arg 56 in procaspase-9 prodomain. Interestingly, this presumption was perfectly supported by molecular docking studies on leonurine or **ZYZ-488**/Apaf-1 CARD complex using Schrodinger software suite 2015. As depicted in Fig. 7A, in the binding mode predicted by docking, the electropositive guanidyl group of leonurine forms four hydrogen bonds (2.1 Å, 2.1 Å, 2.0 Å, 1.6 Å) with the electronegative side chains of Asp 27 and Glu 40. The hydrogen bonds network is of great importance in the binding of the Apaf-1 CARD/procaspase-9 prodomain complex^18^. In addition, the 3-OH of leonurine could accept two hydrogen bonds (2.4 Å, 1.9 Å) from the side chain of Arg 44 in Apaf-1 CARD. As in the case of **ZYZ-488**/Apaf-1 CARD (Fig. 7B), more favorable interactions could be found. Similarly, four hydrogen bonds (2.3 Å, 2.2 Å, 2.1 Å, 1.7 Å) between the guanidyl group of **ZYZ-488** and the two acidic residues of Apaf-1 CARD (Asp 27 and Glu 40) could be observed. Apart from that, **ZYZ-488** anchors the glucuronic acid moiety in a positive center formed by Lys 21, Arg 44, and Arg 52 of Apaf-1 CARD. Hence, the binding of **ZYZ-488** are more stable. These results indicate that leonurine and **ZYZ-488** could occupy the caspase recruitment site of Apaf-1, thus block its interaction with procaspase-9, further resulting in the observed anti-apoptotic effect *in vitro*. Moreover, the more significant anti-apoptotic effect of **ZYZ-488** as compared with leonurine may be attributed to the additional interactions of the glucuronic acid moiety binding to the positive center in Apaf-1 CARD.

### Identification of Molecular target of compound ZYZ-488

**Figure 8:**
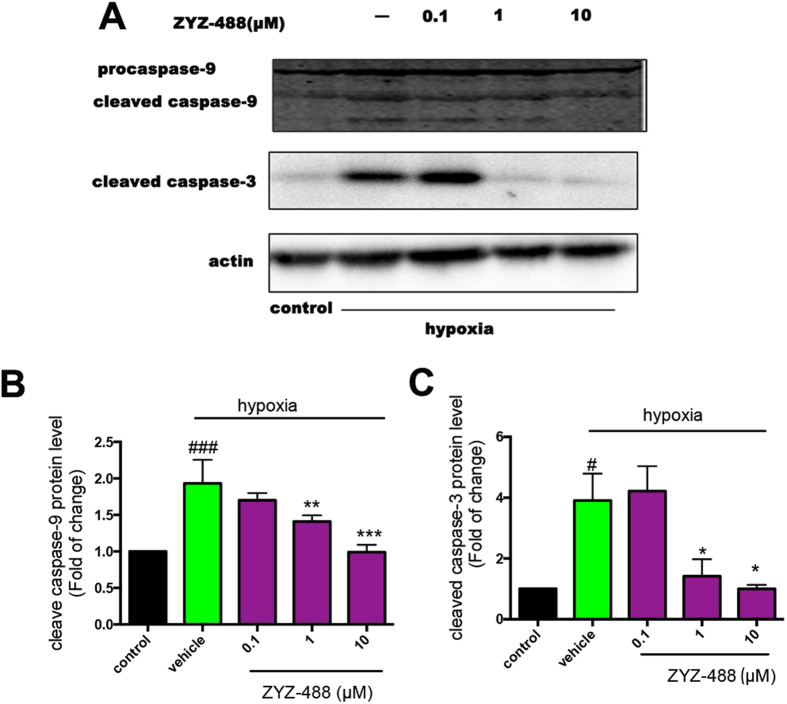
Effects of compound ZYZ-488 on activation of caspase-9 and caspase-3. H9c2 cells were exposed to normoxia or hypoxia for 12 h with or without **ZYZ-488** treatment. (**A**) Representative Western blot showed hypoxia induced Apaf-1-mediated activation of procaspase-9 and procaspase-3 was inhibited by **ZYZ-488**. Bar graphs represent the quantitative difference in protein level of caspase-9 (**B**) and caspase-3 (**C**). ^###^*P* < 0.001, ^#^*P* < 0.05 versus control group. ^*^P < 0.05; ^**^P < 0.01; ^***^P < 0.001 versus vehicle group. Data are the mean ± S.E.M of results from at least three independent experiments.

Apaf-1 plays a critical role in apoptosis by binding to and activating procaspase-9. Activation of procaspase-9 initiates a protease cascade that subsequently activates downstream procaspase-3, leading to the cleavage of target proteins and then orderly demise of the cell^21^. Based on the interaction between **ZYZ-488** and Apaf-1 described as above, we infer that **ZYZ-488** could bind to Apaf-1 at the Apaf-1/procaspase-9 binding site, antagonizing the activation of procaspase-9. To validate this deduction, the effect of compound **ZYZ-488** on the Apaf-1’s activation of procaspase-9 and downstream signaling pathways were determined in H9c2 cells by western blot analysis. The results showed that hypoxia promoted activation of procaspase-9 compared with control group, this activation were remarkably inhibited in a dose-dependent manner after the treatment of compound **ZYZ-488** (Fig. 8 and Fig. S10). Consistently, the downstream procaspase-3’s activation were obviously declined with the exposure to compound **ZYZ-488**.

To exclude the possibility that **ZYZ-488** exerts its inhibitory effects through down regulation of Apaf-1 expression, we examined Apaf-1 expression after **ZYZ-488** treatment in normoxic and hypoxic condition (Figs S7 and S12). As shown in Fig. S7, compound **ZYZ-488** did not affect the expression of Apaf-1 compared with vehicle group.

### The protective effect of ZYZ-488 requires the presence of Apaf-1 in H9c2 cells

**Figure 9:**
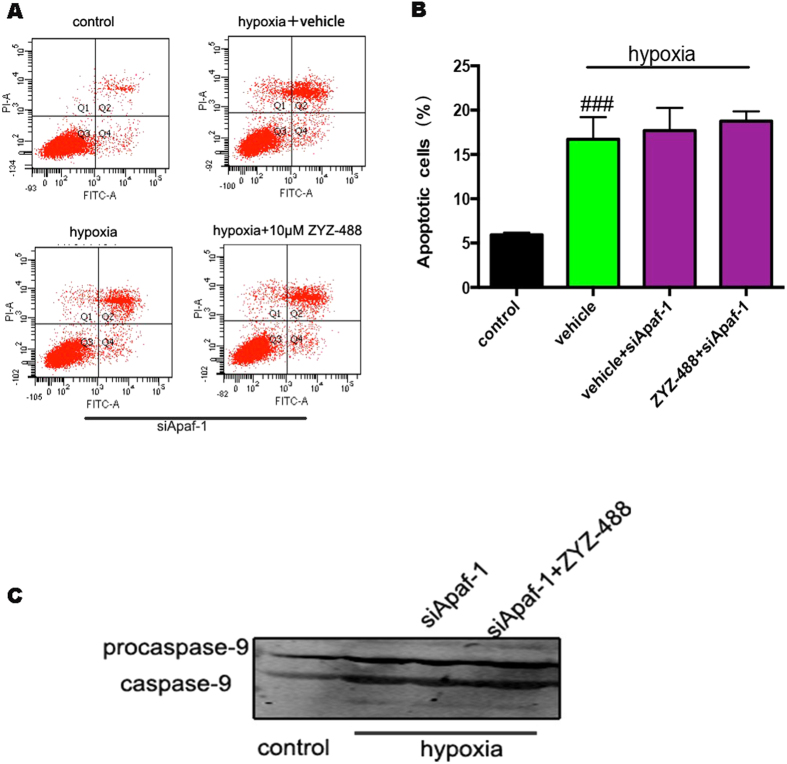
Apaf-1 is required for the inhibitory activity of ZYZ-488. Apaf-1 was silenced in H9c2 cells by Apaf-1 siRNA. (**A**) Apoptosis fraction of hypoxia-induced H9c2 cells were measured in presence or absence of **ZYZ-488** treatment. (**B**) Percentage of apoptotic cardiomyocytes were analyzed. ^###^*P* < 0.001 versus control group. Data are the mean ± S.E.M of results from at least three independent experiments. (**C**) Representative Western blot showed caspase-9 activity for control or Apaf-1 (siApaf-1) silencing in the presence or absence of **ZYZ-488** treatment.

In order to explore if all the observed effects were dependent on the direct Apaf-1 inhibition, the studies on target specificity was performed in cellular models by siRNA based approaches. The silencing effect towards Apaf-1 was observed with three different siRNA (Fig. S9). The siRNA79389 was selected for the silence of Apaf-1. The apoptotic cell counts were significantly increased in hypoxia condition [(16.73 ± 3.02)%] compared with control group [(5.93 ± 0.25)%] (Fig. 9A,B). However, we discovered that hypoxia-induced cell apoptosis can’t be inhibited by **ZYZ-488** in Apaf-1 siRNA-based knock down H9c2 cells. As showed in Fig. 9B that the apoptotic H9c2 cells induced by hopoxia with **ZYZ-488** treatment [(18.77 ± 1.11)%] display no significant change with vehicle group [(17.70 ± 2.56)%]. Results present the inhibition of apoptosis is lost when Apaf-1 is silenced. These results correlated with the western blot of caspase-9 (Fig. 9C and Fig. S11), suggesting that the inhibitory towards caspase-9 activation of **ZYZ-488** dependent on the levels of Apaf-1 in the cell. Taken together, we propose that **ZYZ-488** could competitively bind to Apaf-1 against procaspase-9, and thus tampering the activation of proprocaspase-9.

## Discussion

Apaf-1 is a rather large protein (130 kDa) that carries multiple functional domains, namely an N-terminal caspase activation recruitment domain (CARD), the CED4 homology domain (which include the dATP/ATP-binding motif), as well as 13 C-terminal WD-40 repeats, which allow for its interaction with Cyt c and for its oligomerization. Apaf-1 is the molecular core of the apoptosome, a multiproteic complex mediating the so-called mitochondrial pathway of cell death^22^. However, there is few reports on Apaf-1 pharmacological inhibition. Nelson Chau^23^ reported Aven an endogenous protein has the ability to complex with Apaf-1 and inhibit self-association of Apaf-1 raises the possibility that Aven may impair oligomerization of Apaf-1. Malet^24^ *et al*. identified a novel class of trialkylglycine-based molecules that binds reversibly to Apaf-1 in a cytochrome *c* noncompetitive manner and precludes the recruitment and activation of procaspase-9. QM31^25^ was reported as a chemical inhibitor of Apaf-1 exerts mitochondria-protective functions while the direct interaction between QM31 and Apaf-1 was not clearly defined. Mar^9^ *et al*. demonstrated SVT016426 avoid apoptosis, and the effects were dependent on Apaf-1 inhibition through the lost of effect in siRNA knockdown cells. They proposed that the SVT family of Apaf-1 inhibitors binds to Apaf-1 at the CARD-NOD interface or at the reported *d*ATP binding site in the NOD domain.

Here, we have successfully synthesized an active metabolite of **LEO**, compound **ZYZ-488**. Pharmacological evaluation showed **ZYZ-488** possessed potent cardioprotective effects, which was elicited through its suppression of hypoxia-induced apoptosis. It is important to note that **ZYZ-488** has stronger anti-apoptotic effects than its parent drug **LEO**. Different from the inhibitors mentioned above, molecular docking results suggest the similarity between **ZYZ-488** and the arginine of procaspase-9 prodomain makes **ZYZ-488** capable of inhibiting Apaf-1 recruitment and activation of procaspase-9 as a mimic of procaspase-9 which could competitively bind to Apaf-1 against procaspase-9, and thus tampering the activation of proprocaspase-9 and procaspase-3. Most importantly, compound **ZYZ-488** did not show any noticeable effect in H9c2 cells where Apaf-1 was knocked down, which demonstrate the anti-apoptotic effect of **ZYZ-488** indeed dependent on Apaf-1 inhibition. In summary, we propose compound **ZYZ-488** as a novel competitive, synthetic small molecule inhibitor of Apaf-1 acting through interaction with Apaf-1 in procaspase-9 binding site.

## Methods

### General

All chemical reagents and solvents were purchased from commercial sources and used without further purification. Thin-layer chromatography (TLC) was performed on silica gel plates. Column chromatography were performed using silica gel (Hailang, Qingdao), 200–300 mesh and MCI gel CHP20P (Mitsubishi chemical,Japan). NMR spectra were recorded employing a 400 or 600 MHz Bruker spectrometer. Mass spectral data was collected on a HP5973 N analytical mass spectrometer. HRMS data were determined on an IonSpec 4.7 T FTMS instrument. All reagents of cell culture were purchased from Sigma (St. Louis, MO) unless otherwise stated. The following substances Dulbecco’s Modified Eagle medium (DMEM) and calf serum were purchased from Invitrogen Inc. (MD, USA). All antibodies used for western blotting in this study were from Cell Signaling Technology, Inc. (MA, US). The kits for, Lactic Dehydrogenase (LDH), Creatine Kinase (CK), were obtained from Jiancheng Bioengineering Institute (Nanjing, China). The kit for cell counting kit-8, Hoechst staining was obtained from Beyotime Bioengineering Institute (Lianyungang, China). AnnexinV-FITC/PI kit were obtained from Becton, Dickinson and Company (USA).

The purities of all newly synthesized compounds were analyzed by HPLC, with over 95% of purity. Analytical HPLC was performed on a Waters 600E system chromatograph equipped with photodiode array detector using a MG-C18 5 μm 250 mm 4.6 mm column (reverse phase) to detect the purity of the products. The mobile phase was a gradient of 30% methanol flow rate of 1.0 mL/min.

#### (2*S*,3*R*,4*S*,5*S*,6*S*)-2-(4-((*Z*)-8-((*tert*-butoxycarbonyl)amino)-12,12-dimethyl-10-oxo-2,11-dioxa-7,9-diazatridec-8-en-1-oyl)-2,6-dimethoxyphenoxy)-6-(methoxycarbonyl)tetrahydro-2*H*-pyran-3,4,5-triyl triacetate (7)

Bromo-sugar **5** (27.6 g, 0.0693 mol) and **6** (35.4 g, 0.0693 mol) was dissolved in CHCl_3_ (25 mL). To the above stirred solution Tetrabutyl ammonium bromide (22.4 g, 0.0693 mol) and K_2_CO_3_ (0.2 mol/L, 26 mL) were added. The reaction mixture was heated at 45 °C for 12 h. After completion of the reaction, CHCl_3_ (25 mL) was added, and then washed by 1N HCl, saturated NaHCO_3_ (2 × 25 mL) and brine (2 × 25 mL). The organic layer was separated, dried over Na_2_SO_4_, the solvent was evaporated. The residue was purified by column chromatography on silica gel to afford compound **7** 11.2 g (40% yield). ^1^H NMR (400 MHz, CDCl_3_): δ 6.93 (s, 2H), 5.60 (t, 1H, J = 7.44 Hz), 5.47–5.21 (m, 3H), 4.34 (t, 2H, J = 6.3 Hz), 4.12 (q, 1H, J = 8 Hz), 3.88 (s, 6H), 3.68 (s, 3H), 3.52 (dd, 2H, J1 = 8 Hz, J2 = 12 Hz), 2.05 (s, 9H), 1.83 (m, 2H), 1.74 (m, 2H), 1.49 (s, 9H), 1.48 (s, 9H). ^13^C NMR (400 MHz, CDCl_3_): *δ* 170.24, 169.40, 169.19, 167.08, 165.93, 155.99, 153.25, 152.73, 152.73, 137.84, 126.67, 106.93, 100.33, 83.47, 79.88, 77.39, 77.07, 76.75, 72.75, 72.40, 71.86, 56.43, 52.69, 40.65, 28.21, 26.15, 25.82, 20.65. HRMS (ESI): calculated for C_37_H_53_N_3_O_18_ [M + H]^+^ 828.3397, found 828.3404.

#### (2*S*,3*R*,4*S*,5*S*,6*S*)-2-(4-((4-guanidinobutoxy)carbonyl)-2,6-dimethoxyphenoxy)-6-(methoxycarbonyl)tetrahydro-2*H*-pyran-3,4,5-triyl triacetate (8)

Compound **7** (1.2 g, 1.45 mmol) was dissolved in 10 mL CH_2_Cl_2_:TFA (1:1,v/v) solution and stirred for 12 h. Saturated NaHCO_3_ solution (100 mL) was added to the reaction mixture and stirred for 30 min. Then extracted with CH_2_Cl_2_, The organic layer was separated, dried over Na_2_SO_4_, the solvent was evaporated to provide yellow solid compound **8** (yield 92%) ^1^H NMR (400 MHz, D_2_O): *δ* 7.20 (s, 2H), 5.47-5.21 (m, 3H), 5.04 (d, 1H, *J* = 6.7 Hz), 4.34 (d, 2H, *J* = 4.9 Hz), 3.71 (s, 6H), 3.68 (s, 3H), 3.25–.01 (m, 4H), 2.75 (t, 2H, *J* = 6 Hz), 2.05 (s, 9H),1.61 (dt, 4H, *J*_1_ = 32 Hz, *J*_2_ = 4 Hz),^13^C NMR (150 MHz, CDOD_3_): *δ* 173.75, 172.83, 168.44, 168.37, 159.64, 149.93, 141.04, 128.57, 109.40, 105.29, 103.73, 78.4, 78.11, 76.31, 74.06, 66.03, 58.06, 58.00, 57.84, 43.04, 27.96, 27.56 24.48, 22.03. HRMS (ESI): calculated for C_27_H_37_N_3_O_14_ [M + H]^+^ 628.2348, found 628.2357.

#### (2*S*,3*S*,4*S*,5*R*,6*R*)-6-(4-((4-guanidinobutoxy)carbonyl)-2,6-dihydroxyphenoxy)-3,4,5-trihydroxytetrahydro-2*H*-pyran-2-carboxylic acid (ZYZ-488)

Compund **8** (6.27 g, 10 mmol) was dissolved in 50 mL CH_2_Cl_2_:MeOH(1:9, v/v) solution and cooled to 0 °C, and then fresh guadine in ethanol was added and stirred for 2 h, immediately there was yellow solid deposited. The solid was filtrated and then purified by column chromatography on MCI CHP20 gel to provide **ZYZ-488** (508 mg, yield 11%). ^1^H NMR (600 MHz, D_2_O): *δ* 6.93 (s, 2H), 5.04 (d, 1H, *J* = 11.6 Hz), 4.04 (d, 2H, *J* = 6.9 Hz), 3.71 (s, 6H), 3.59-3.47 (m, 4H), 3.09 (t, 2H, *J* = 6.43 Hz), 1.61 (dt, 4H, *J*_1_ = 5.6 Hz, *J*_2_  = 13 Hz). ^13^C NMR (150 MHz, C): *δ* 175.24, 167.11, 156.65, 151.93, 137.51, 125.69, 106.66, 102.29, 77.12, 75.47, 73.56, 71.80, 65.43, 56.05, 40.67, 25.01, 24.66. HRMS (ESI) calculated for C_20_H_29_N_3_O_11_ [M + H]^+^ 488.1875, found 488.187

### Cell lines and induction of hypoxia

H9c2 rat ventricular cardiomyocytes (ATCC, Manassas, VA) were cultured in Dulbecco’s modified Eagle medium (Gibco-Invitrogen, Carlsbad, CA) supplemented with 10% fetal bovine serum, in tissue culture flasks at 37 °C in a humidified atmosphere of 5% CO_2_. The cells were fed every 2–3 days and subcultured once they reached 70%–80% confluence. Hypoxia was induced based on the technique described by Rakhit *et al*.^26^. All culture plates, excluding the normoxic control, were placed in an ischemia solution (composition (in mmol/L): NaCl 116; KCl 50; CaCl_2_ 1.8; MgCl_2_·6H_2_O 2; NaHCO_3_ 26; NaH_2_PO_4_·2H_2_O 1) in an anaerobic chamber (BD Diagnostics System, Maryland, NJ, USA) maintained at 37 °C with a humidified atmosphere of 5% CO_2_, 10% H_2_ and 85% N_2_. Chambers were sealed before incubation at 37 °C for 5 h. Normoxic incubation of myocytes in serum-free DMEM was conducted in a water-jacked incubator gassed with 95% air and 5% CO_2_ at 37 °C for the same length of time. LipofectamineTM 2000 (Invitrogen) was used according to the manufacturer’s instructions to transfect H9c2 cells and Apaf-1 siRNA (GenePharma, Shanghai).

### Cell Survival Assay

The effects of **ZYZ-488** and **LEO** on cardiomyocyte viability were obtained using cell counting kit-8 (CCK8) assay. Briefly, H9c2 cells were seeded on 96-well plates (approximately 8000 cells/well) in Dulbecco’s modified Eagle medium culture medium and maintained in regular growth medium for 2 days. The culture medium was then changed to ischemia solution with or without drugs, placed in an anaerobic chamber for hypoxia induction, then incubated at 37 °C for 12 h. After adding 10 μL of the CCK-8 reagent to each well, the wells were incubated for 1 h at 37 °C and 5% CO_2_. The absorbance of each well were measured at 450 nm in a microtiter plate reader.

### Determination of Lactate Dehydrogenase in Culture Medium

Creatine Kinase leakage into the culture medium was analyzed using Creatine Kinase Kit (Biotime, Haimen, China). CK release was expressed as a fold relative to the activity in normoxic cells.

### Assessment of Apoptosis by Flow Cytometry

Apoptosis and necrosis were identified by means of double fluorescence staining with annexin V-propidium iodide. Cardiomyocytes (1 × 10^5^ cells per sample) were loaded with 5 μL PI and 10 μL Annexin V-FITC (BD,USA) at room temperature for 15 minutes in the dark. Flow cytometric analysis was performed using a flow cytometer (Becton- Dickinson, Mountain View, CA), after of Annexin-PI labeling.

### Fluorescent staining of nuclei

H9c2 nuclei were stained with chromatin dye (Hoechst 33258). Briefly, cells were fixed with 3.7% paraformaldehyde for 10 minutes at room temperature, washed twice with phosphate buffered solution (PBS), and incubated with 10 μM hoechst 33258 in PBS at room temperature for 30 min. After three washes, cells were observed under a confocal microscope (LSM 510, ZEISS).

### Molecular Simulation and Physiochemical Properties calculationh

Docking studies were carried out using Schrodinger modeling suite^19^. The X-ray crystallographic structure of Apaf-1^18^ (PDB code: 1CY5) was taken from Protein Data Bank. Crystal water molecules were deleted. All missing hydrogen atoms were added by standard protein preparation protocol within Maestro followed by energy minimization using OPLS 2005 force field to optimize all hydrogen bonding networks. The structures of leonurine and **ZYZ-488** were prepared with the default p*K*_a_ range of 7.0 ± 2.0 in the ligand preparation program LigPrep^27^. Docking of both compounds were carried out using Glide^28^ with standard precision protocol (Small-Molecule Drug Discovery Suite 2015-4: Glide, version 6.9, Schrödinger, LLC, New York, NY, 2015). The vander Waals radii of nonpolar atoms for each of the ligands were scaled by a factor of 0.8 to account for structure variability to specific ligand binding.

### Western blot analysis

Cultured H9c2 cells were harvested by scraping and centrifugation, washed with PBS, and re-suspended in RIPA buffer. Soluble proteins were collected by centrifugation at 12,000 g. Protein lysates were subjected to 10% and 12% SDS-PAGE and transferred onto a NC membrane (Millipore Corporation). After blocking with 5% skim milk, the membranes were incubated with the respective primary antibodies (caspase-3, 1:1000; caspase-9, 1:1000, Apaf-1, 1:1000 Cell Signaling Technology, USA) in Tris-buffered saline (TBS) containing 0.1% Tween-20 overnight at 4 °C. The membranes were then incubated with the appropriate secondary horseradish peroxidase-conjugated IgG antibodies at a 1:5000 dilution (Proteintech Group Inc., USA). Immunoreactive proteins were then visualized using ECL. The signals were quantified by densitometry using a Western blotting detection system (Bio-Rad Laboratories, Inc, USA). Actin served as the loading control.

### Statistical analysis

Values are shown as means ± S.E.M of at least 3 independent preparations. The significance of differences between groups was evaluated with t tests or one-way ANOVA followed by the Dunnett’s multiple comparison tests. Values of P < 0.05 were considered significant. All statistics were carried out using PRISM 6.0 for MAC.

## Additional Information

**How to cite this article**: Wang, Y. *et al*. The discovery of a novel inhibitor of apoptotic protease activating factor-1 (Apaf-1) for ischemic heart: synthesis, activity and target identification. *Sci. Rep.* **6**, 29820; doi: 10.1038/srep29820 (2016).

## Supplementary Material

Supplementary Information

## Figures and Tables

**Figure 1:**
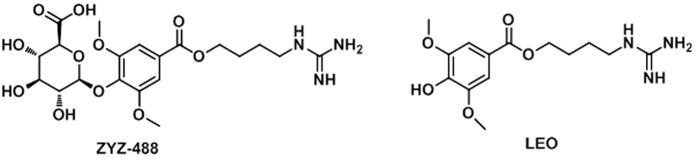
Chemical structures of ZYZ-488 and Leonurine.
