# Supplementary material for: The discovery of a novel inhibitor of apoptotic protease activating factor-1 (Apaf-1) for ischemic heart: synthesis, activity and target identification
Source: Sci Rep. 2016 Jul 22;6:29820. doi: 10.1038/srep29820 (PMC4957240; doi:10.1038/srep29820)

Supporting Information

The discovery of a novel inhibitor of apoptotic protease activating factor-1 (Apaf-1) for ischemic heart: synthesis, activity and target identification

Ying Wang[a], Yang Cao[b], Qing Zhu[c], Xianfeng Gu[b]*, Yi-Zhun Zhu[a,d]*

[a] *Department of Pharmacology, School of Pharmacy and Institute of Biomedical Sciences, Fudan University, Shanghai 201203, China*

[b] *Department of Medicinal Chemistry, School of Pharmacy, Fudan University, Shanghai 201203, China*

[c] *School of Pharmacy, Nantong University, Nantong, 226001, China*

[d]Department of Pharmacology, LYY [f]School of Medicine, National University of Singapore

Table of contents

Table S1. The predicted target candidates for kinases of compound ZYZ-488 using PharmMapper server[1] 1
Figure S1. ^{1}H NMR(600MHz,D_2O) for compound ZYZ-488........ 3
Figure S2. ^{13}C NMR(150MHz,D_2O) for compound ZYZ-488 4
Figure S3. ^{1}H NMR(400MHz, $CDCl_3$) for compound 7........ 5
Figure S4. ^{13}C NMR(100MHz, $CDCl_3$) for compound 7........ 6
Figure S5. ^{1}H NMR (400MHz, D_2O) for compound 8........ 7
Figure S6. ^{13}C NMR (150MHz, $CDOD_3$) for compound 8........ 8
Figure S7. The effect of ZYZ-488 on expression of Apaf-1 under normoxic or hypoxic condition. 9
Figure S8. Effect of ZYZ-488 on CHK-2 phosphorylation and activation of caspase-8.10
Figure S9. Silencing effect of siApaf-1........10

Table S1. The predicted target candidates for kinases of compound ZYZ-488 using PharmMapper server[1]

Note that in silico targets screening was performed within an in-house pharmacophore databae, named PharmTarget DB, which contains more than 7000 receptor-based pharmacophore models, and only those kinases at the top 300 (about 9%) in the ranked list were extracted and summarized.

Rank[a]	PDB	Target	Fit	Normalized	Molecular and pharmacophore

	ID	Name	Score	Fit Score	Aligned Model[b]
Compound ZYZ-488					
22	1Z6T	APAF-1	4.476	0.4069	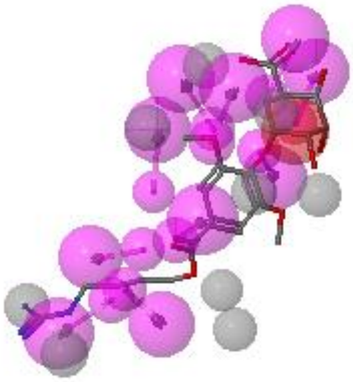

[a] The ranked list of hit target pharmacophore models, which are sorted by fit score in descending order;

[b] The molecule and pharmacophore were aligned by PharmMapper, and the molecule-pharmacophore alignment poses were displayed via a modified version of Jmol applet. The pharmacophore features are schemed by color: Hydrophobic, cyan; Positive, blue; Negative, red; Donor, green, Acceptor, magenta; Aromatic, yellow;

[c] The result for compound 2 can be freely accessed and downloaded from the PharmMapper server via

http://59.78.96.61/pharmmapper/result.php?job_id=14099133002

1. Liu, X., et al., *PharmMapper server: a web server for potential drug target identification using pharmacophore mapping approach.* Nucleic acids research, 2010. **38**(suppl 2): p. W609-W614.

Figure S1. ^{1}H NMR(600MHz,D_2O) for compound ZYZ-488

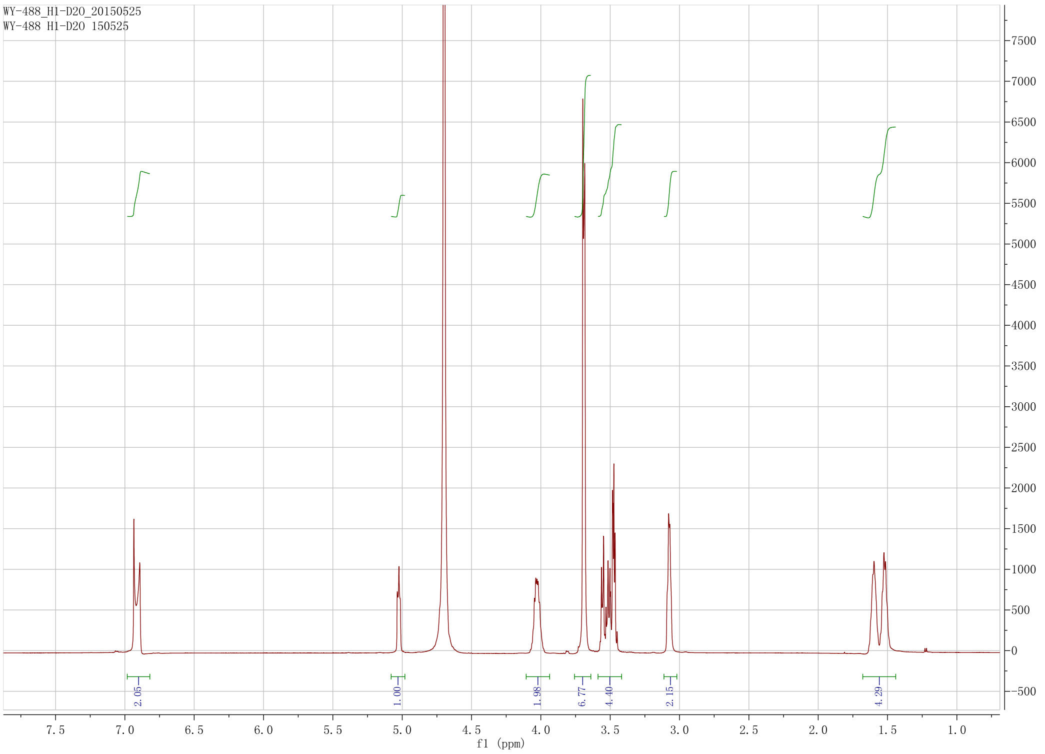

Figure S2. ^{13}C NMR(150MHz,D_2O) for compound ZYZ-488

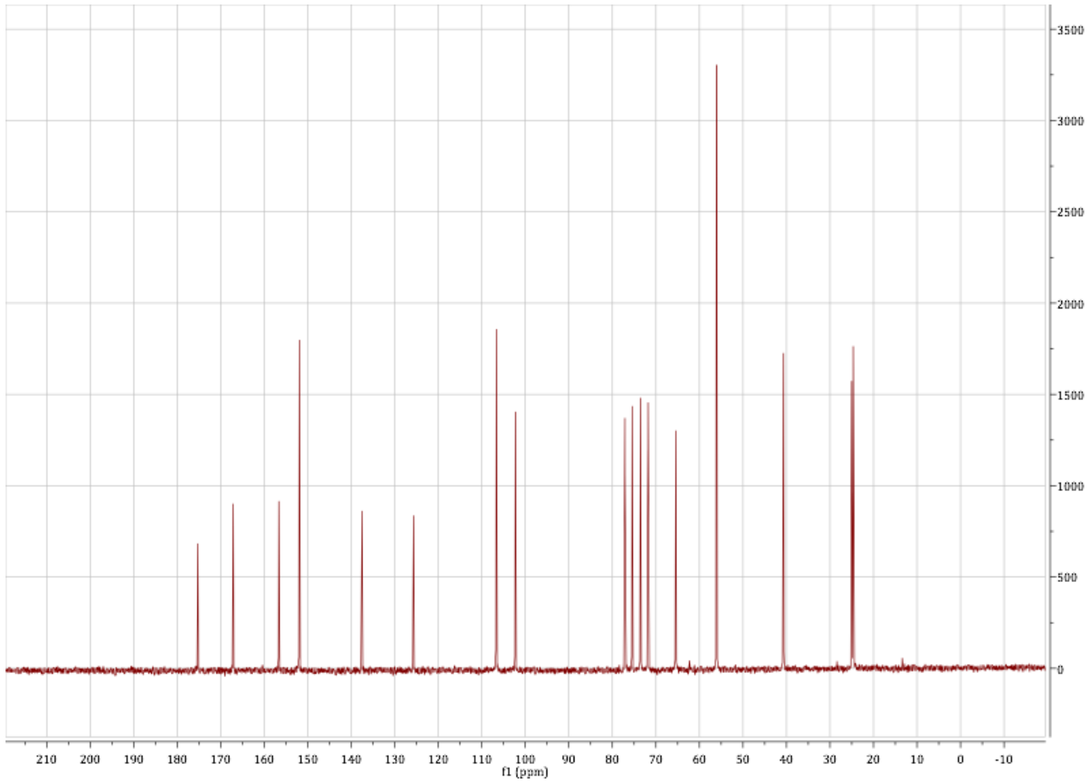

Figure S3. ^{1}**H NMR(400MHz,** $CDCl_3$**) for compound 7**

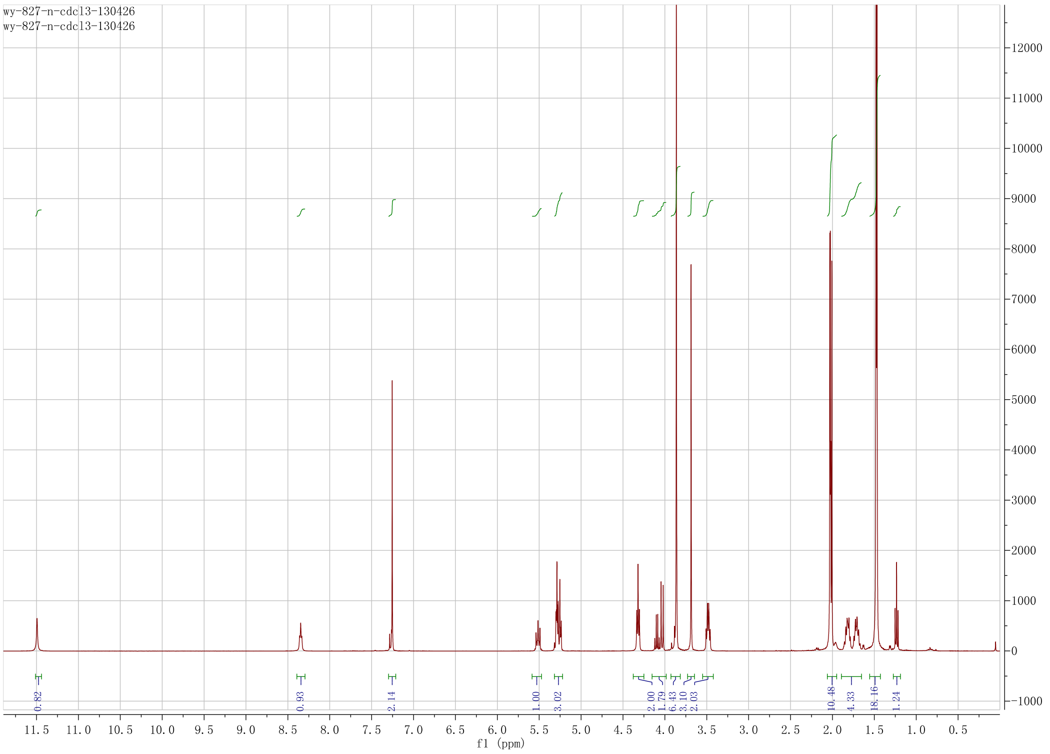

Figure S4. ^{13}C NMR(100MHz, $CDCl_3$) for compound 7

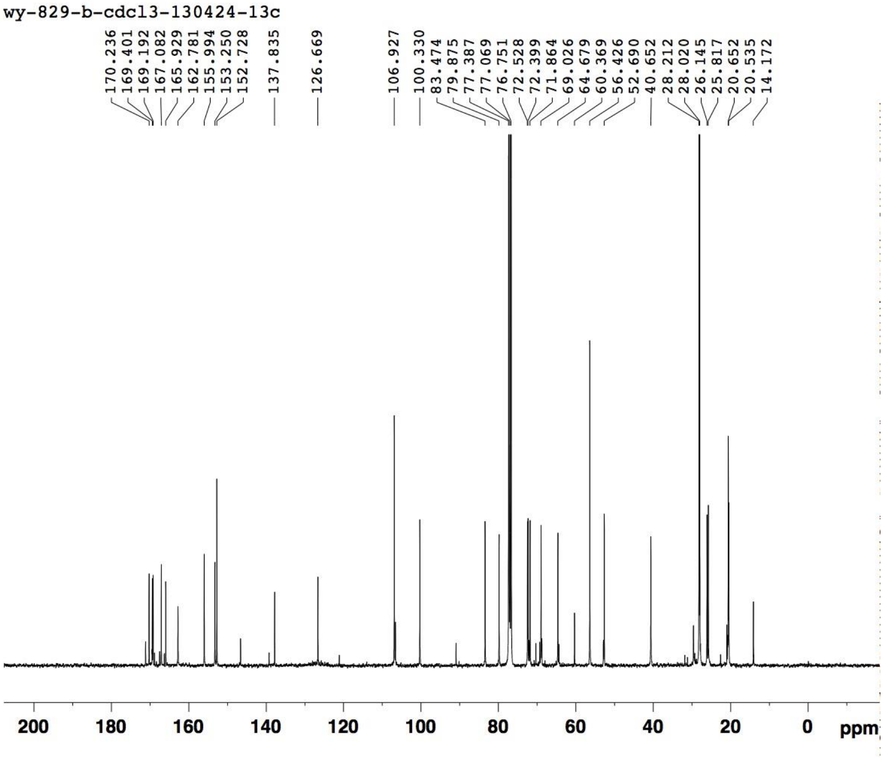

Figure S5. ^{1}H NMR (400MHz, D_2O) for compound 8

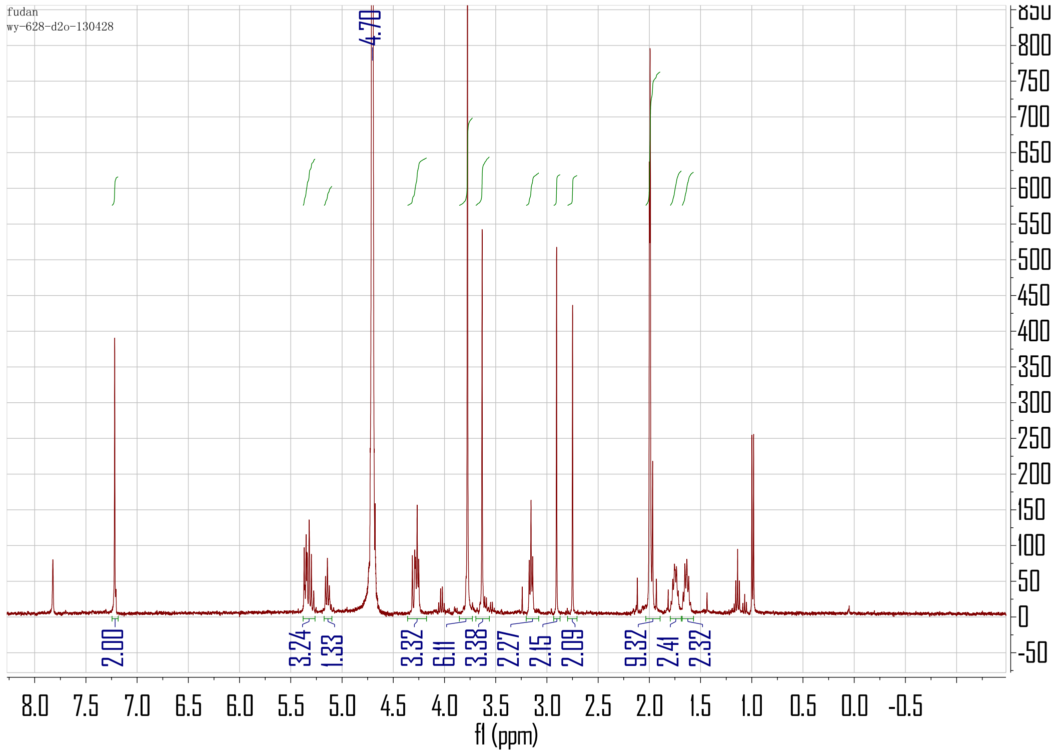

Figure S6. ^{13}C NMR (150MHz, $CDOD_3$) for compound 8

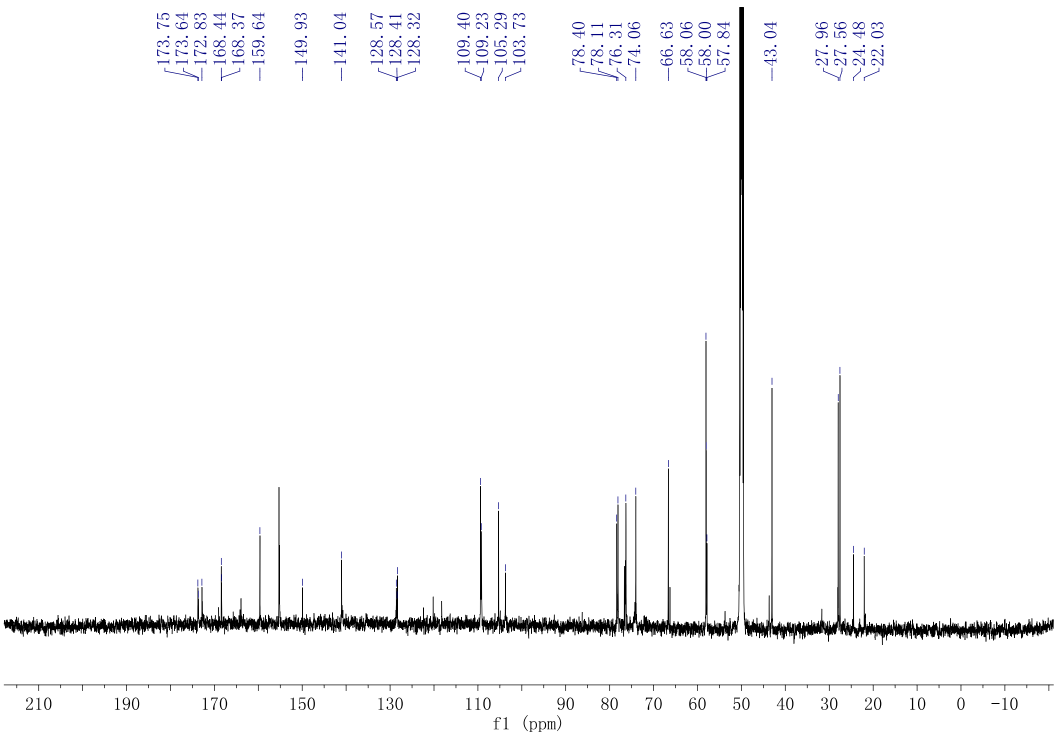

Figure S7. The effect of ZYZ-488 on expression of Apaf-1 under normoxic or hypoxic condition.

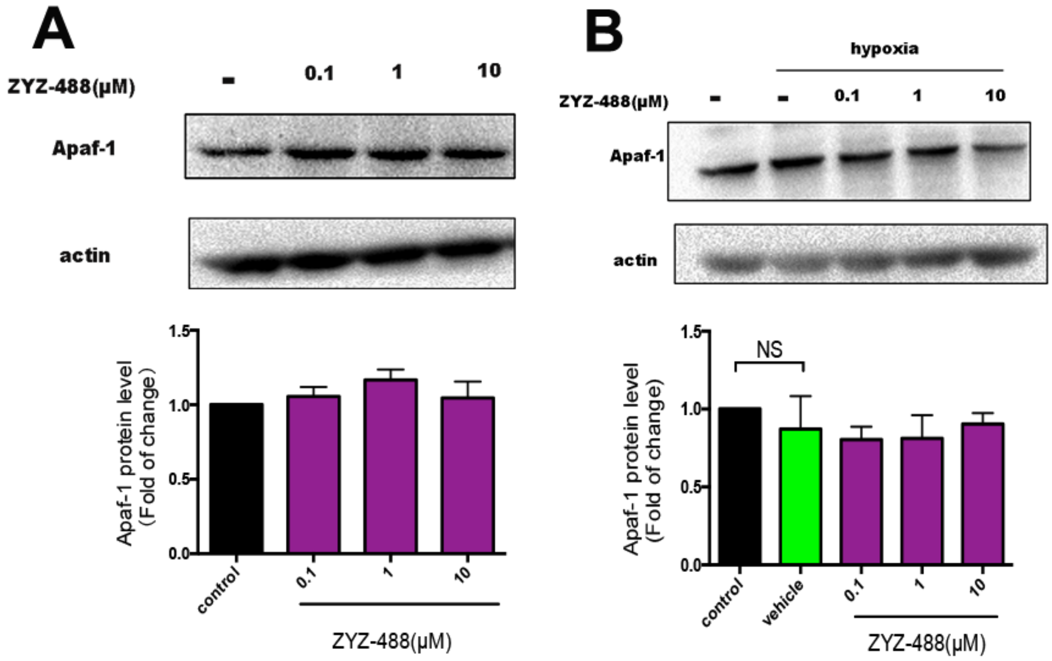

Figure S7. The effect of **ZYZ-488** on expression of Apaf-1 under normoxic or hypoxic condition. H9c2 cells were incubated with indicated concentrations of **ZYZ-488** for 12 h. Then Apaf-1 protein level under normoxic condition was analyzed by Western blot (A). H9c2 cells were culture under hypoxic condition for another 12h to analyze the Apaf-1 protein level under hypoxic condition. Data are the mean ±S.E.M of results from at least three independent experiments.

Figure S8. Effect of ZYZ-488 on CHK-2 phosphorylation and activation of caspase-8.

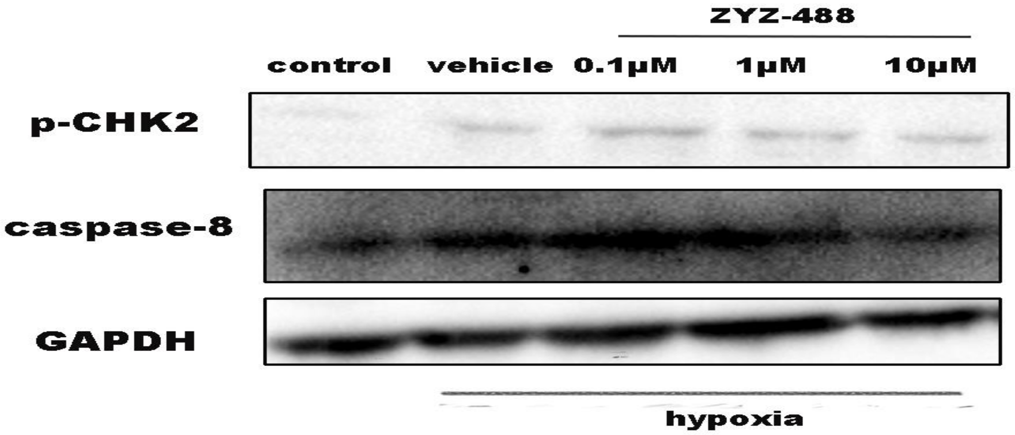

Figure S9. Silencing effect of siApaf-1

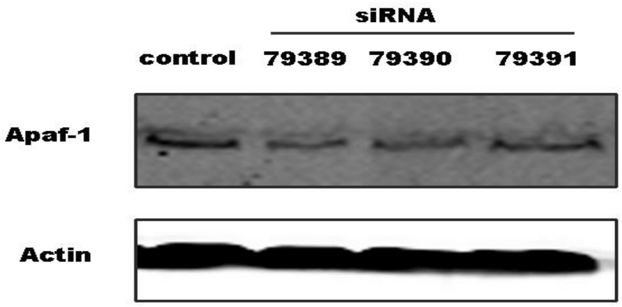

Figure S10. The full-length blots of figure 8.

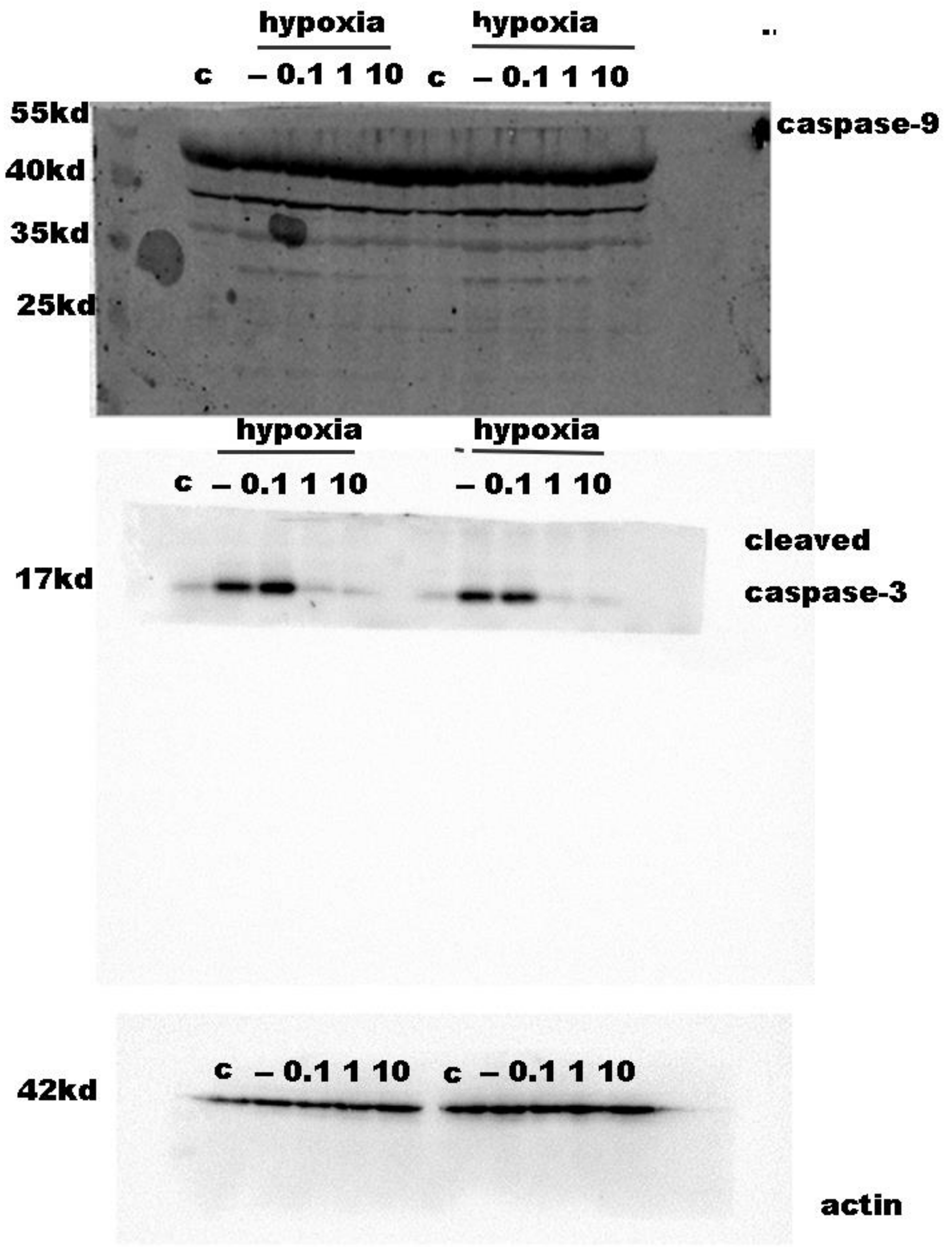

Figure S11. The full-length blots of figure 9.

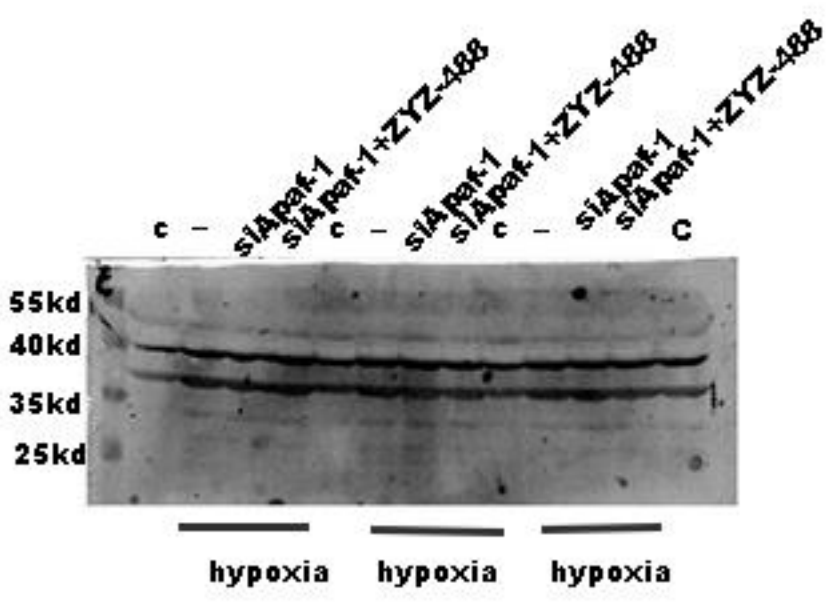

Figure S12. The full-length blots of figure S7 and S8.

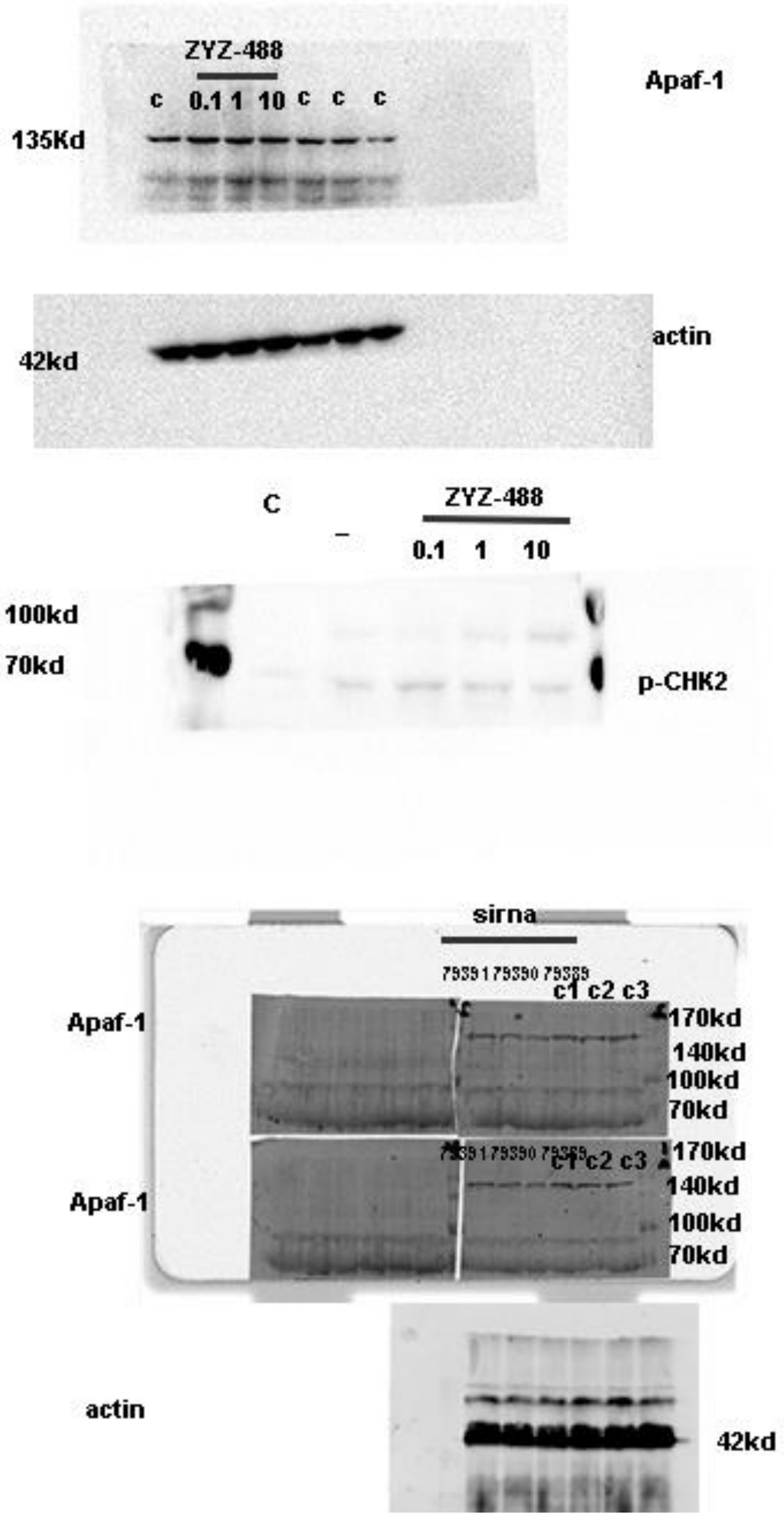

Supplement: Supplementary Information [file srep29820-s1.pdf]
